# PATRIC, the bacterial bioinformatics database and analysis resource

**DOI:** 10.1093/nar/gkt1099

**Published:** 2013-11-12

**Authors:** Alice R. Wattam, David Abraham, Oral Dalay, Terry L. Disz, Timothy Driscoll, Joseph L. Gabbard, Joseph J. Gillespie, Roger Gough, Deborah Hix, Ronald Kenyon, Dustin Machi, Chunhong Mao, Eric K. Nordberg, Robert Olson, Ross Overbeek, Gordon D. Pusch, Maulik Shukla, Julie Schulman, Rick L. Stevens, Daniel E. Sullivan, Veronika Vonstein, Andrew Warren, Rebecca Will, Meredith J.C. Wilson, Hyun Seung Yoo, Chengdong Zhang, Yan Zhang, Bruno W. Sobral

**Affiliations:** ^1^Virginia Bioinformatics Institute, Virginia Tech, Blacksburg, VA 24060, USA, ^2^Computation Institute, University of Chicago, Chicago, IL 60637, USA, ^3^Mathematics and Computer Science Division, Argonne National Laboratory, Argonne, IL 60637, USA, ^4^Grado Department of Industrial & Systems Engineering, Virginia Tech, Blacksburg, VA 24060, USA, ^5^Department of Microbiology and Immunology, University of Maryland School of Medicine, Baltimore, MD 21201, USA, ^6^Fellowship for Interpretation of Genomes, Burr Ridge, IL 60527, USA, ^7^Computing, Environment, and Life Sciences, Argonne National Laboratory, Argonne, IL 60637, USA and ^8^Nestlé Institute of Health Sciences SA, Campus EPFL, Quartier de L'innovation, Lausanne, Switzerland

## Abstract

The Pathosystems Resource Integration Center (PATRIC) is the all-bacterial Bioinformatics Resource Center (BRC) (http://www.patricbrc.org). A joint effort by two of the original National Institute of Allergy and Infectious Diseases-funded BRCs, PATRIC provides researchers with an online resource that stores and integrates a variety of data types [e.g. genomics, transcriptomics, protein–protein interactions (PPIs), three-dimensional protein structures and sequence typing data] and associated metadata. Datatypes are summarized for individual genomes and across taxonomic levels. All genomes in PATRIC, currently more than 10 000, are consistently annotated using RAST, the Rapid Annotations using Subsystems Technology. Summaries of different data types are also provided for individual genes, where comparisons of different annotations are available, and also include available transcriptomic data. PATRIC provides a variety of ways for researchers to find data of interest and a private workspace where they can store both genomic and gene associations, and their own private data. Both private and public data can be analyzed together using a suite of tools to perform comparative genomic or transcriptomic analysis. PATRIC also includes integrated information related to disease and PPIs. All the data and integrated analysis and visualization tools are freely available. This manuscript describes updates to the PATRIC since its initial report in the 2007 NAR Database Issue.

## INTRODUCTION

In 2002, the National Institute of Allergy and Infectious Diseases (NIAID) developed a strategic plan for Biodefense research that defined the ‘Priority Pathogens’ and developed a subsequent watch list of genera, categorized as A, B and C priority microbial pathogens (http://www.niaid.nih.gov/topics/biodefenserelated/biodefense/pages/cata.aspx). This initiative outlined the scope of biodefense research to understand the biology of these organisms and to develop new diagnostics, treatments and vaccines to treat them. In 2004, NIAID provided funding for eight Bioinformatics Resource Centers (BRCs) to provide scientists with genomics and related data on these organisms (1) and also on invertebrate vectors that transmit them. Two of these original eight BRCs were the Pathosystems Resource Integration Center (PATRIC) (2) and the National Microbial Pathogen Data Resource (NMPDR) (3). PATRIC originally stored and integrated data on eight different bacterial and viral pathogen groups, whereas NMPDR covered five of the bacterial genera on the watch list. In 2009, NIAID reorganized the BRC program through a competitive renewal, consolidating into four BRCs, each one with a discrete yet all-encompassing organismal focus: bacteria, viruses, eukaryotic pathogens and invertebrate vectors. A single exception, the Influenza Resource Database, was initiated to specifically focus on the influenza virus. PATRIC was awarded the bacterial BRC and collaborated with the NMPDR team to produce one of the most comprehensive data analysis resources available for bacteria (http://www.patricbrc.org), providing researchers with thousands of consistently annotated bacterial genomes, integrating the related ‘omics data and providing a suite of analysis tools to support infectious disease research. Here, we give a detailed description of the new features added during the transformation of PATRIC from a resource that began with eight genomes and limited integration to its current capacity of more than 10 000 genomes, with a variety of other integrated data types and improved analysis capabilities.

## NEW DATA

In its current incarnation, PATRIC hosts data from all the NIAID priority pathogenic bacteria, which include 22 genera. However, to understand the virulence and pathogenicity that sets these bacteria apart from their nonpathogenic relatives, it also includes data for all publicly available assembled bacterial genomes sequences. As of September 2013, there are more than 10 000 bacterial genomes available in PATRIC with projections of more than 15 000 by the end of the year. Along with the genomes and their annotations, PATRIC also provides a variety of integrated ‘omics datasets. A summary of the integrated data at the level of Bacteria and across the target genera is illustrated (see Table 1), and descriptions of individual data types are provided below.

**Table 1.** Targeted pathogens with data types currently available in PATRIC

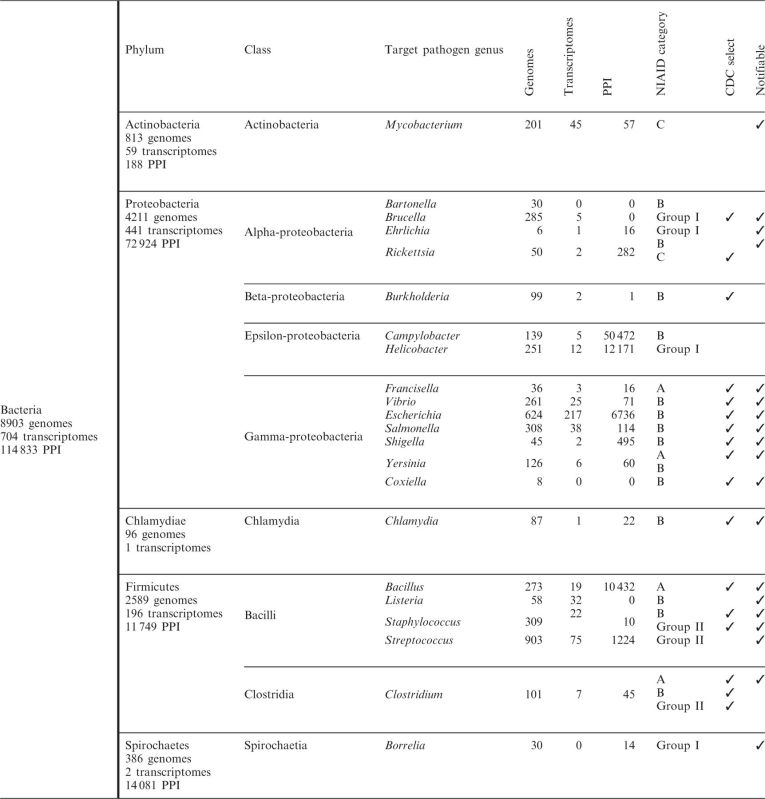

Group I—pathogens newly recognized in the past two decades.

Group II—re-emerging pathogens.

### Genomic data

Every month PATRIC collects genomes from GenBank (4) and RefSeq (5), using automated scripts for data collection and incorporation. Genomes from collaborators are also uploaded upon request.

Every genome available at PATRIC is annotated using Rapid Annotations using Subsystems Technology (RAST) (6), an end-user genome annotation service that was developed under the NMPDR project and currently annotates more than 4000 genomes per month. With more than 12 000 users, RAST is the most widely used and highly cited automated microbial annotation system and has annotated more than 100 000 microbial genomes as of this writing. It uses a common controlled vocabulary shared with SEED and ModelSEED (http://www.theseed.org/wiki/Main_Page), and also KBase (http://kbase.science.energy.gov/) to support inter-comparison of annotations and functional content. In addition, PATRIC also maintains the original annotations from GenBank to allow users to explore and compare differences. However, having every genome consistently annotated with the same technology and terminology makes PATRIC truly unique in that researchers can make comparisons across taxonomic boundaries without wondering if the differences they are observing are related to different annotation sources. As part of genome annotation, proteins are mapped to protein families called FIGfams, which enable comparative genomic analysis at PATRIC. FIGfams are isofunctional homologs, with each FIGfam containing a set of proteins that are ‘end-to-end homologous and share a common function’. (7) They are constructed from careful manual curation of subsystems and automated analysis of closely related strains and are based on sequence similarity over the entire protein length, and on the conserved genomic context.

#### Taxonomic data

**Figure 1.**
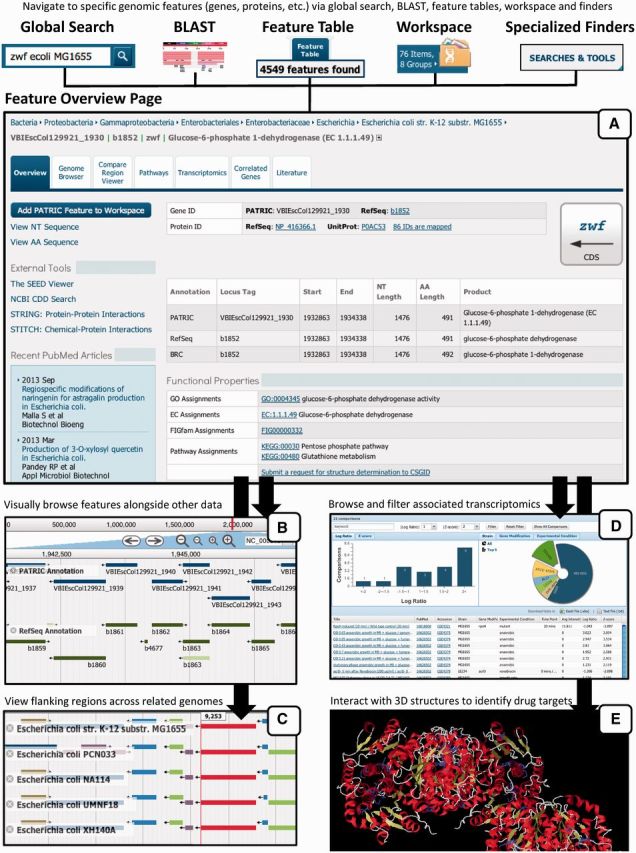
Integration and displays of different data types at the level of the gene. PATRIC provides various methods to search for individual genes that lead to a landing page where data are summarized **(A)** and also provides access to a variety of gene-centric tools and visualizations including a genome browser **(B)**, the Compare Region viewer **(C)**, a summary and filter mechanism for gene expression data **(D)** and a link to any structural data that might exist (D).

All bacterial data available at PATRIC are mapped to NCBI Taxonomy (8), which uses the hierarchical structure of the taxonomy classification to summarize and collate data consistently at all taxonomic levels. Levels range from super kingdom of all bacteria, to phylum, class, order, family, genus, species, subspecies, strain, isolate and individual genomes. PATRIC parses out taxonomic information for all the genomes and synchronizes the taxonomy classification with NCBI every month, incorporating any changes. Data are summarized across all taxonomic levels on special landing pages. Each taxon page summarizes all genomes and features contained within that taxon. For example, the ‘All Bacteria’ taxon page includes all PATRIC genomes and features, whereas the ‘*Mycobacterium’* taxon page includes only those genomes and features contained within that genus. Data are also summarized at the genome level, which identifies all the integrated data for an individual strain. A third summary of data occurs at the gene level (Figure 1A) where the physical characteristics of a gene, its functional properties, available experimental data and associated publications are presented. This provides researchers with a fully integrated summary of information in a single gene page.

#### Genome metadata

Equally important to genome annotations are the metadata associated with genomes, which provide invaluable information such as isolation source, geographic location, year of isolation and the host and/or environment from which the sample was collected. Metadata from clinical isolates can include information on antibiotic resistance, patient health and symptoms. With the deluge of available and projected genomic data, this type of meta-information is essential for sorting through and finding genomes of interest. PATRIC automatically gathers genome metadata from a variety of sources, including the original GenBank records, BioProject and BioSample (9) by parsing more than 60 different metadata fields, and retrieving information from free-text genome descriptions and organizing it into a structured format. In addition, PATRIC works closely with the NIAID funded Genomic Sequencing Centers for Infectious Diseases to standardize metadata. The collected data are reviewed and refined by human curators for accuracy, consistency and completeness.

### Transcriptomics data

High throughput technologies, such as microarrays and RNA-Seq, allow simultaneous measurement of expression of thousands of genes on a genome-wide scale. There are thousands of gene expression datasets in the public repositories such as GEO (10) and ArrayExpress (11), and this number continues to grow. These data provide a wealth of information about the expression of genes under different conditions, but are inaccessible for most biologists due to heterogeneity in the use of technologies, platforms, data analysis protocols and data and metadata formats.

In partnership with GenExpDB (http://genexpdb.ou.edu/main/), we have developed a system where new microarray gene datasets are collected from GEO every month. Platform annotations, data and metadata are automatically collected and then manually reviewed. This manual curation includes a review of the experiment description and the related publication to understand experimental design; combines data from replicates; and creates pair-wise comparisons or contrasts as described in the publication to identify differential gene expression, data normalization and log-transformation. Using the above process, 704 transcriptomic datasets across 65 bacterial genera (Table 1) have been incorporated into PATRIC from GEO; this number will continue to grow every month. At present, all gene expression data integrated into PATRIC are from experiments examining differential gene expression.

### Protein–protein interactions

PATRIC has incorporated ∼1300 experimentally characterized host–pathogen protein–protein interactions (PPIs) and more than 100 000 bacterial PPIs from a number of public repositories, including IntAct (12), BIND (13), DIP (14) and MINT (15). These data are obtained by mining the public repositories of interaction data found in the PSICQUIC (16) registry. New interactions are retrieved and added every month using automated scripts and the most recent release of the tool includes host–pathogen interactions from BIND, BioGRID (17), DIP, IntAct and MINT. PATRIC also provides interactions within and among bacterial genera using this process.

### Protein 3D structure data

Currently, there are more than 33 000 experimentally solved bacterial protein structures available in various public data repositories such as the PDB (18) and NCBI MMDB (19). The protein three-dimensional (3D) structures are mapped to the corresponding PATRIC bacterial proteins using ID mapping. The 3D structures from these two repositories are retrieved in real-time using APIs, mapped to the corresponding bacterial proteins in PATRIC and displayed on the website using the Jmol (20) viewer (Figure 1E). In addition, NIAID has funded two Structural Genomics Centers for Infectious Diseases that apply high-throughput structural biology techniques to characterize 3D structures of targeted proteins from NIAID priority pathogens (21,22). These centers have produced 3D structures of hundreds of proteins related to virulence factors, essential enzymes and potential drug and vaccine targets. In addition to incorporating the structures, PATRIC also provides links to the corresponding protein pages on the structural genomics centers websites where information about target selection criteria and related clones are available. PATRIC also provides the ability to easily submit community target requests to the centers in cases where no structure currently exists, prefilling ID and sequence information.

### Sequence typing data

Bacterial typing methods based on phenotype (biotype, serotype and phage type) have traditionally been used for epidemiological studies and outbreak investigations (23). When these typing methods are included in the GenBank or RefSeq annotation data, they are integrated into PATRIC by separate, searchable metadata fields making bacterial genomes searchable within and between typing methods. The PATRIC Multi-Locus Sequence Typing (MLST) pipeline extracts MLST sequence types from bacterial genomes using the PubMLST API for reference MLST profiles (24). This allows genomes in PATRIC to be searchable by the sequence-based typing method that has become the gold standard of global infectious disease epidemiology.

### Data integration

PATRIC integrates data across sources, data types, molecular entities and organisms. Data are collected and integrated from public repositories such as NCBI, the United States Department of Agriculture, the Centers for Disease Control (CDC) and other NIAID-funded projects, such as the System Biology for Infectious Disease centers, as well as from studies carried out by individual investigators. There are distinct procedures in place to work with different types of data providers, with data automatically collected from public repositories.

**Figure 2.**
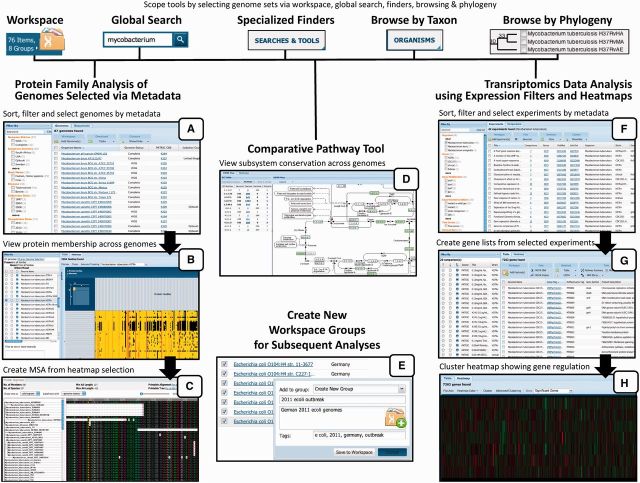
Comparative analysis tools at PATRIC. Researchers can use a variety of searches and browsers to find, select and save data to a private workspace, from which they deploy a number of comparative genomic analysis tools, including the Protein Family Sorter that has metadata filtering **(A)**, heatmap summary of protein families **(B)**, gene trees and multiple sequence alignments **(C)**. The Comparative Pathway tool summarizes data from selected genomes across KEGG pathways **(D)**. The Transcriptomic Data Analysis tool has both an expression and metadata filtering mechanism **(F)** that creates a gene list where data can be filtered to show up or down regulation **(G)** from which a heatmap view with advanced clustering capabilities of the data can be deployed **(H)**. All analysis tools allow researchers to collect and refine data in their private workspace **(E)**.

Much of this integration centers on genomes and genes. Biological entities such as genes, RNAs, proteins, pathways and other data are readily linked to genomes. Genomes also provide an anchor for linking pathogens, hosts and vectors to molecular data. Metadata about genomes are used to allow users to search, filter and collect data of interest (Figure 2A). In addition, molecular level experimental ‘omics data, such as transcriptomics, proteomics, PPIs and protein structures, are likewise integrated with the genes and proteins, and then propagated to similar genes in other closely related genomes via protein families.

### Searching/finding data in PATRIC

As the number of genomes being sequenced increases, so does the difficulty in finding genomes of interest and pertinent associated data. Although PATRIC contains thousands of comparable genomes, there are mechanisms in place that allow researchers to find genomes of interest including single specific genomes, whole genome sets within certain taxa, sets of genomes that share common metadata, and even closely related sets of genomes based on phylogeny. Researchers can locate genomes of interest using a variety of means including a global search deployed from the main navigation bar, phylogenetic trees, genome/gene lists, genome/feature finder tools and metadata filters (Figure 2). Once selected, genomes can be saved as a group to a private workspace. Researchers can also easily select all genomes at any taxonomic level and analyze them together.

In addition, both NCBI- and PATRIC-centric versions of BLAST allow users to find genes or genomes that match specific sequence criteria. In addition, PATRIC has an ID mapping tool that leverages UniProt’s ID mappings, which allows researchers to map identifiers from a variety of resources (including RefSeq, GenBank, KEGG, UniProt, etc.) to the corresponding PATRIC identifiers.

### Private workspace

Once researchers have found genomes or data of interest, they can create groups based upon their selections. These user-specified groups of genomes are an important precursor to many of PATRIC’s sophisticated analysis tools. To save these groups, PATRIC provides registered users with a private workspace, functioning as a digital laboratory notebook (Figure 2E). The workspace includes the ability to form and retain groups of genomes or proteins for later reference. This is especially useful as researchers sometimes take considerable efforts to locate genomes or genes that they are interested in comparing, and would like to be able to return where they left off in the analysis without starting from scratch. These saved datasets are especially useful for researchers working on publications, or merely checking alignments or IDs of data of interest.

### Data downloads

The PATRIC database supports multiple ways to access data, accommodating the needs of both expert and computationally naive users. Researchers have the option of selecting datasets by browsing or searching the database through the website user interface that supports both ad hoc queries and directed navigation methods. Results may be saved to data files and downloaded as needed. In addition to APIs, PATRIC also provides an FTP service for bulk data downloads.

## NEW ANALYTICAL CAPABILITIES

PATRIC is designed primarily to allow experimental biologists to view and analyze data in an easy and efficient manner. The processes, tools and viewers used to find and analyze the data integrated within PATRIC are described below, as well as the process for researchers to upload and explore their private data with PATRIC.

### Genomic analysis at PATRIC

PATRIC’s compilation of all public bacterial genomes and their consistent annotation using RAST provides a powerful platform for comparative genomic analysis. The Protein Family Sorter tool at PATRIC allows users to select a set of genomes of interest (maximum up to 400 genomes) and examine distribution of protein families across the genomes (Figure 2A), commonly referred to as the ‘pan genome’, which in this case refers to the superset of proteins found in all selected genomes. This tool provides various filtering options to quickly locate protein families that are conserved across all the genomes (‘core genome’), conserved only in a subset of the selected genomes (‘accessory genome’) or that match a specified function. A tabular view shows protein families matching filtering criteria and an interactive heatmap viewer (Figure 2B) provides a bird’s-eye (‘pan genome’) view of the distribution of the protein families across multiple genomes, with clustering and anchoring functions to show relative conservation of synteny and identify lateral transfers.

An integral part of genomic analysis is phylogeny, and PATRIC provides Order-level phylogenetic trees. The pipeline used was initially developed during the first incarnation of PATRIC (25–27) and uses a series of tools that builds trees based on shared protein families (27).

PATRIC’s Comparative Pathway tool is also based on the RAST annotations. It allows researchers to identify a set of metabolic pathways based on taxonomy, EC number, pathway ID, pathway name and/or specific annotation type. The data are mapped to and summarized on pathway maps (Figure 2D) from the Kyoto Encyclopedia of Genes and Genomes, commonly known as KEGG (28). This tool also provides a table of unique pathways that match the search criteria (i.e. the genomes or proteins chosen by the researcher, or at any taxonomic level) from which researchers can select specific pathways of interest and view a KEGG Map, or on a heatmap view that summarizes the data, including presence/absence of individual EC numbers within the selected genomes.

PATRIC provides the ability to compare RAST and RefSeq annotations using the Genome Browser (Figure 1B) built on JBrowse (29). Researchers can upload and view their own annotations and compare them with PATRIC annotations in the same browser. It also allows researchers to compare gene neighborhoods using the Compare Region Viewer (Figure 1C) and to construct gene trees and/or alignments with the Multiple Sequence Alignment tool (Figure 2C).

### Transcriptomic analysis at PATRIC

PATRIC integrates transcriptomic and genomic data, allowing researchers to explore gene expression data across species, within a specific genome, or for a gene of interest. As of September 2013, 704 transcriptomic experiments across 27 bacterial genera have been incorporated with manually curated data and metadata. All datasets are listed and summarized on the appropriate taxon landing pages along with experiment metadata, such as organism, strain, gene mutations and experimental conditions. The available functionality includes a suite of integrated tools to explore, visualize and compare a large number of published transcriptomics datasets. PATRIC also allows researchers to upload and analyze their own gene expression data in their private workspace and compare it to existing data. Metadata-driven filtering allows users to search for experiments or comparisons of interest (Figure 2F). Interactive gene lists (Figure 2G) and a heatmap with clustering tools (Figure 2H) provide quick access to genes that are differentially expressed or have similar expression patterns across various conditions. These genes can be selected and mapped to metabolic pathways. At the gene level, a graphical summary of metadata for the comparisons relevant to differential expression of the gene (Figure 1D) and summary of genes with correlated expression profiles help assess or hypothesize potential function of a gene, making it a valuable resource for researchers who are interested in studying specific genes or identifying potential function of hypothetical genes.

An RNA-Seq analysis pipeline, called the RNA Rocket, was developed in a cross-BRC project in collaboration with the PATRIC, EuPathDB (30) and Vectorbase (31) teams. This free service is available at the Pathogen Portal (http://www.pathogenportal.org/), where users can upload their RNA-Seq or ChIP-Seq data, align it against a BRC provided genome and generate quantitative transcript profiles using BRC annotations. PATRIC has enabled end-to-end support for transcriptomic data analysis, allowing researchers to stream expression and annotation data from their RNA Rocket workspace to the PATRIC genome browser, as well as viewing differential expressions analysis data in the heat map viewer alongside other integrated transcriptomic experiments.

### Disease-related information at PATRIC

**Figure 3.**
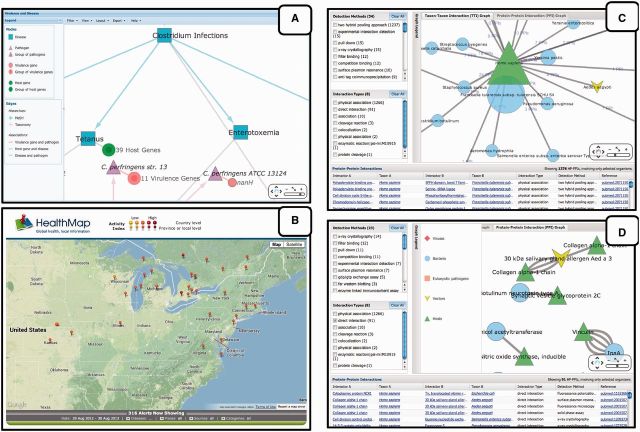
Data integration tools. PATRIC’s Disease View provides an interactive graph **(A)** that summarizes relationships between pathogens, the diseases they cause, and both host and pathogen genes associated with that disease. A collaboration with HealthMap that provides a global summary of geolocation reports showing disease-related information in real-time is also provided **(B)**. The Protein Interaction Gateway collects data from public databases and allows researchers to examine data summarized at the taxon level **(C)**, and also to look at individual PPIs with a sortable grid and node-edged graphs **(D)**.

PATRIC provides Disease View, which provides easy access to integrated information related to hosts, pathogens, infectious diseases and outbreaks by a user-friendly web interface (32). This resource enables the analysis of host–pathogen–disease associations to facilitate the understanding of the disease mechanisms and to assist in the development of diagnostics and therapeutics. This resource associates infectious diseases with corresponding pathogens and provides information on the pathogen, pathogen virulence genes and the genetic and chemical evidence for the human genes that are associated with the diseases. It visualizes these relationships between pathogens, genes and diseases in an interactive graph (Figure 3A) and provides the geolocation reports of associated diseases around the globe in real-time (Figure 3B) via a collaboration with HealthMap (33). Diseases can be viewed at any level of the bacterial taxonomic tree and interactive graphs are provided to enable visual analysis of the host–pathogen–disease associations.

### The Pathogen Interaction Gateway for protein–protein interactions

The Pathogen Interaction Gateway (PIG) (34) provides access to inter- and intra-species PPI data for easy download or visualization. PIG is available currently as a Web application on both the Pathogen Portal (bacteria, viruses, eukaryotic pathogens, hosts and vectors) and PATRIC (bacteria and hosts) websites. Original PPI data are periodically downloaded from public databases that include experimental data (predicted interactions are not included in PIG) and are filtered for taxa of interest, de-duplicated and stored in local databases. PIG allows users to select custom sets of PPIs by taxon (Figure 3C) or keyword combinations, and visualize the results in a coordinated interface that includes a sortable list and interactive PPI node-edge graphs (Figure 3D). Multiple taxa can be visualized simultaneously, enabling the detection of deeper interaction motifs such as host proteins targeted by multiple pathogens, or multiple classes of pathogen (bacterial, viral and eukaryotic), as well as guilt-by-association clues to the function of unknown or hypothetical proteins.

## FUTURE IMPROVEMENTS

Planned improvements in PATRIC include integrating additional data types, annotation enhancements to RAST annotations and expanding the Bring Your Own Data (BYOD) concept.

### New data types

By the end of 2013, PATRIC will incorporate all publicly available proteomics datasets that match certain criteria from PRIDE (35) and from the NIAID-funded Systems Biology Centers. All proteomics datasets will be listed on the appropriate taxon and genome overview pages along with experiment metadata, such as organism, strain, gene mutations and experimental conditions, as PATRIC does for the transcriptomic data. Likewise, our collaborations with individual researchers have expanded our knowledge of metabolomics and phenotype data, and the next phase will include integration of these types of data into PATRIC as well.

In the longer term, PATRIC will continue to be driven by the needs of infectious disease researchers, especially for NIH priority pathogens. We are watching for technologies like metagenomics and metabolic modeling to become more prominent in research communities, and plan to include them when both the data and the associated analysis techniques are more standardized and stable. In addition, as emerging techniques and new types of data drive researchers toward integrated views of both the host or vector and pathogen, this will be used to drive requirements for additional data and capabilities at the website.

### Annotation enhancements

Key improvements relating to annotation of virulence factors and antibiotic resistance genes will be incorporated into RAST and subsequently into PATRIC. Genera-specific annotation of specialty gene sets related to pathogenicity and antibiotic resistance will be carried out using information derived from external sources such as VFDB (36), MvirDB (37), ARDB (38), TBDReaMDB (39) and periodic targeted literature-based curation efforts that will be used to identify gene sets of interest. These gene sets will be used to generate improved protein families that will, in turn, be used to drive the automated RAST annotation process. Periodic re-annotation of the entire PATRIC collection of genomes based on the updated protein families will propagate literature-based improvements to annotations.

### Expanding Bring Your Own Data

PATRIC will launch ‘Bring Your Own Genome’ in 2014 where registered users will be able upload their genomes into their private PATRIC workspace, annotate them using RAST and compare them with the publically available genomes at PATRIC using the comparative analysis tools. PATRIC will also add additional functionality currently available at RAST, including a sequence-based comparison tool. Future plans for BYOD also include support for uploading, validating, viewing and analyzing raw reads (genomic, metagenomics and RNA-Seq), assembled genome sequences and other ‘omics data types, such as proteomics data, transcriptomics data, metabolomics data and growth phenotype data.

## FUNDING

Funding for open access charge: National Institute of Allergy and Infectious Diseases, National Institutes of Health, Department of Health and Human Service [Contract No. HHSN272200900040C].

*Conflict of interest statement*. None declared.
